# Supplementary material for: Fork Reversal Safeguards Epigenetic Inheritance During Replication Stress
Source: Res Sq. 2026 Jan 28:rs.3.rs-8544414. Preprint. [Version 1] doi: 10.21203/rs.3.rs-8544414/v1 (PMC12869582; doi:10.21203/rs.3.rs-8544414/v1)
Supplement: 1 [file NIHPPrs8544414v1-supplement-1.pdf]

## Supplementary Figure Legends

Figure S1 Fork reversal deficiency reduces the nucleosome density on nascent DNA, related to Figure 1

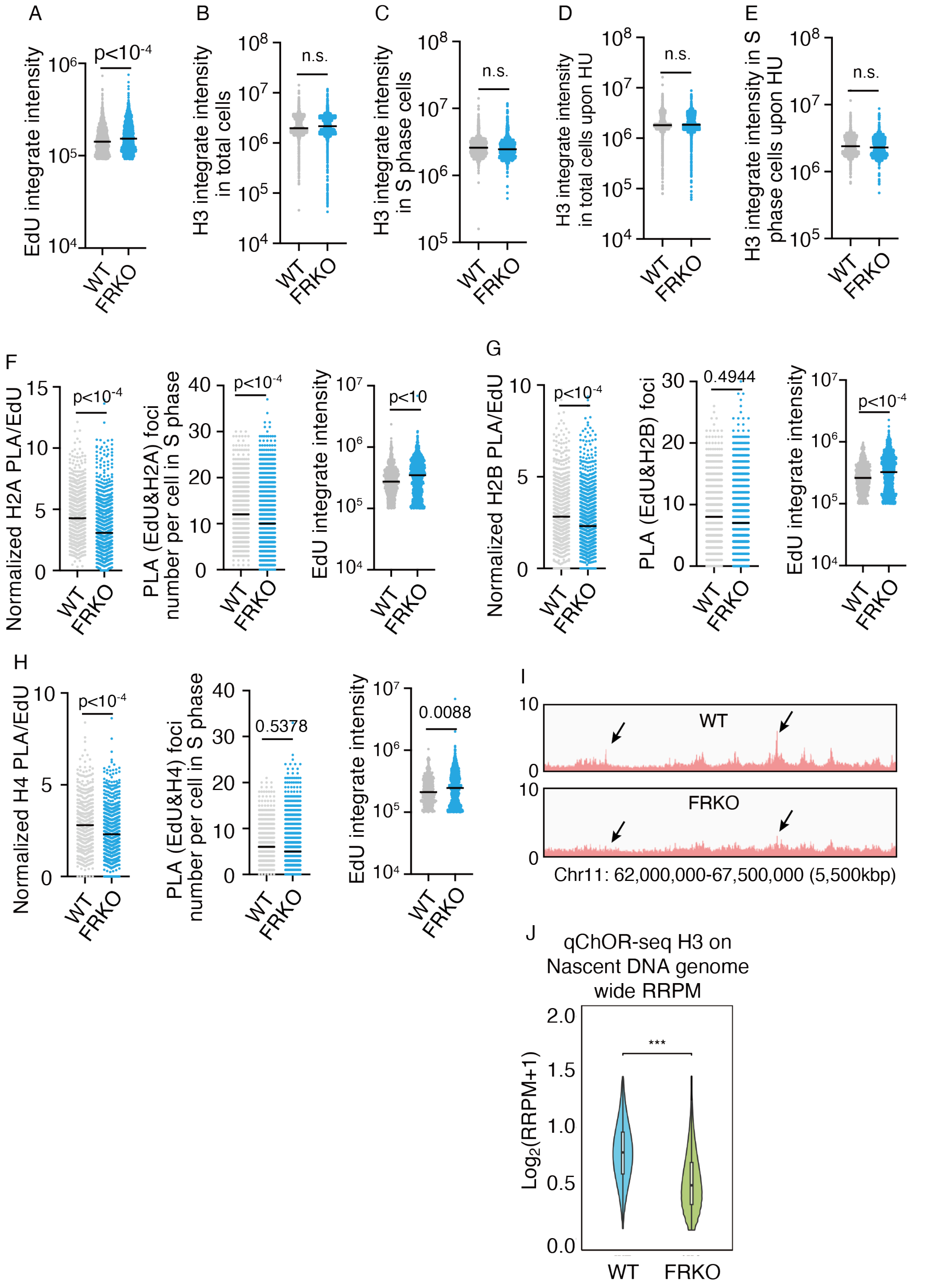

Figure S1. Fork reversal deficiency reduces nucleosome density on nascent DNA (related to Figure 1).

FRKO clone #29 is used in figure S1A-J.

A) EdU incorporation in WT and FRKO cells treated with EdU + HU (as in Fig. 1B). EdU detected by azide-488 click reaction.

B–E) Immunofluorescence for H3 in WT and FRKO cells under indicated conditions. N = 3; p-values from two-tailed unpaired t-test.

F–H) PLA for EdU and H2A (G), H2B (H), and H4 (I). Left: PLA foci/cell; middle: EdU intensity; right: normalized PLA/EdU. $\geq$1,000 cells/sample. N $\geq$ 3.

I) Genome browser views of H3 ChOR-seq from the same experiment in Figure 1E, normalized to reads per million using exogenous spike-in. N = 3.

J) Quantitative ChOR-seq for H3. Average profiles across H3 peaks in WT and FRKO cells, normalized by reference-adjusted RPM using D. melanogaster spike-in. N = 3; Wilcoxon signed-rank test.

Figure S2 Low density of nucleosome is due to fork reversal deficiency rather than individual fork reversal enzyme depletion, related to Figure 1

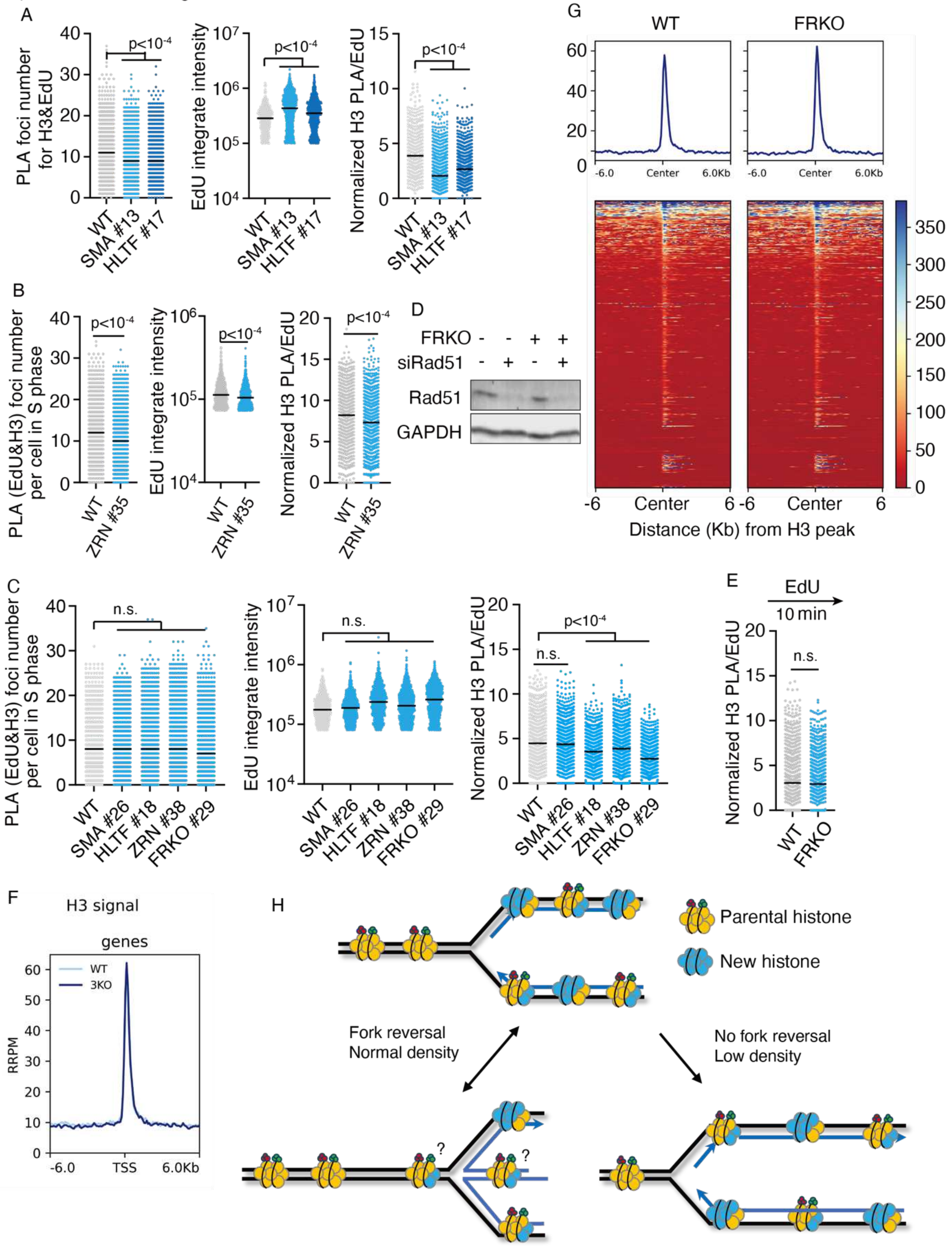

Figure S2. Low nucleosome density phenotype in individual fork reversal enzymes knockout clones of U2OS cell lines (related to Figure 1).

A–C) PLA for EdU and H3 in individual fork reversal enzyme KO clones. N = 3. p-values from unpaired t-test in B) and Kruskal–Wallis test in A) and C).

D) Western blot showing siRAD51 knockdown efficiency.

E) PLA for EdU and H3 in WT and FRKO cells without HU. Cells labeled with EdU (10 min) and harvested for PLA. N = 3; p-values from unpaired t-test.

F) ChOR-seq analysis of H3 occupancy in WT and FRKO cells without HU, normalized to spike-in chromatin. The cells are treated same as in D). N=3.

G) Heatmaps of H3 ChOR-seq signal (same data from F) in unperturbed condition. N = 3.

H) Model schematic: fork reversal prevents low nucleosome density under replication stress.

Figure S3 Fork reversal deficiency leads to parental histone loss, related to Figure 2

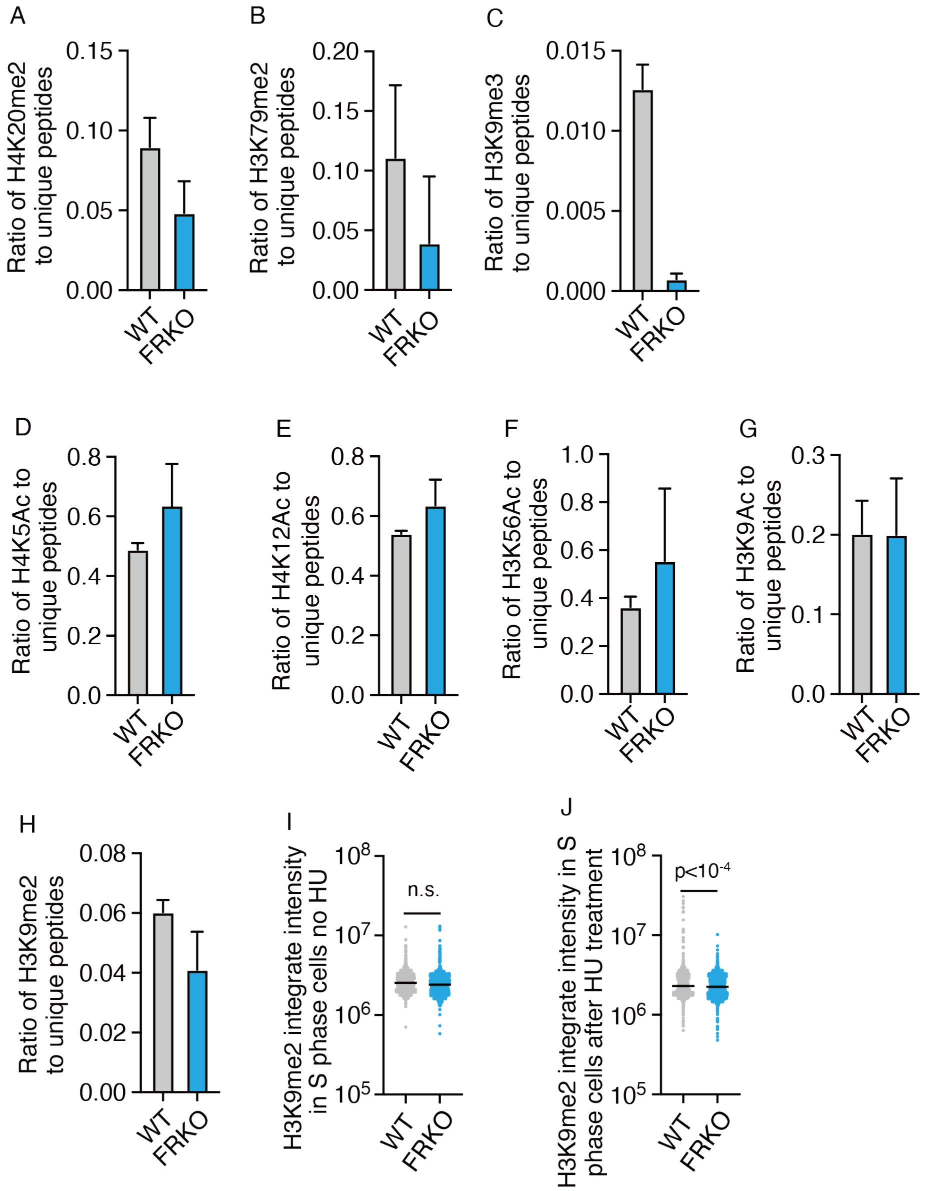


Figure S3. Fork reversal deficiency leads to parental histone loss (related to Figure 2).

A-H) Histone modifications quantified in WT and FRKO cells by iPOND–Propionylation-MS. Modification level equals modified peptide count/unique peptide count. N = 3; error bars are generated from standard deviation.

I-J) Immunofluorescence for H3K9me2 in indicated conditions. N = 3; unpaired t-test.

Figure S4 PrimPol deletion rescues parental histone loss and new histone increase, related to Figure 3

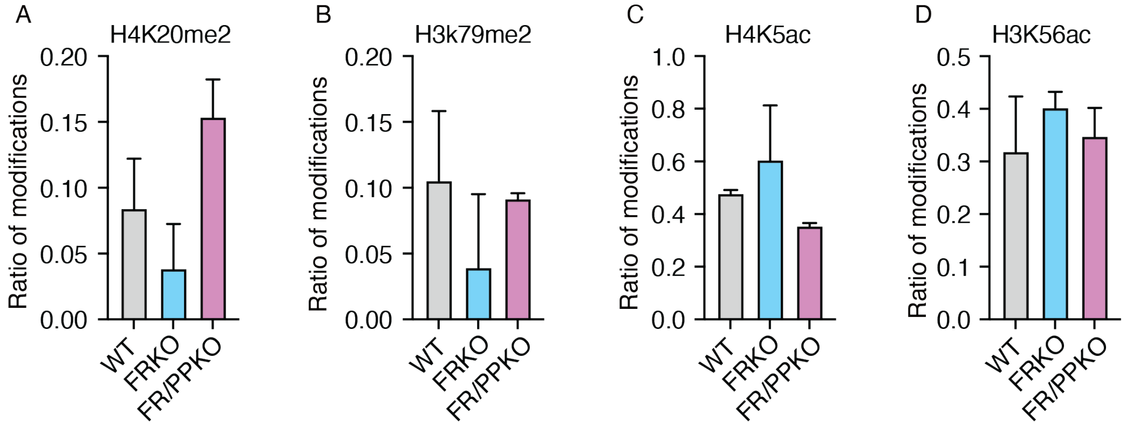


Figure S4. PrimPol depletion rescues parental histone loss and new histone increase, related to Figure 3.

A-D) Histone modifications quantified in WT, FRKO, and FR/PPKO cells by iPOND–Propionylation-MS. Modification level equals modified peptide count/unique peptide count. N = 3; error bars are generated from standard deviation.

Figure S5 Single-stranded DNA gaps loosen the nucleosome assembly on nascent DNA in cells, related to Figure 4

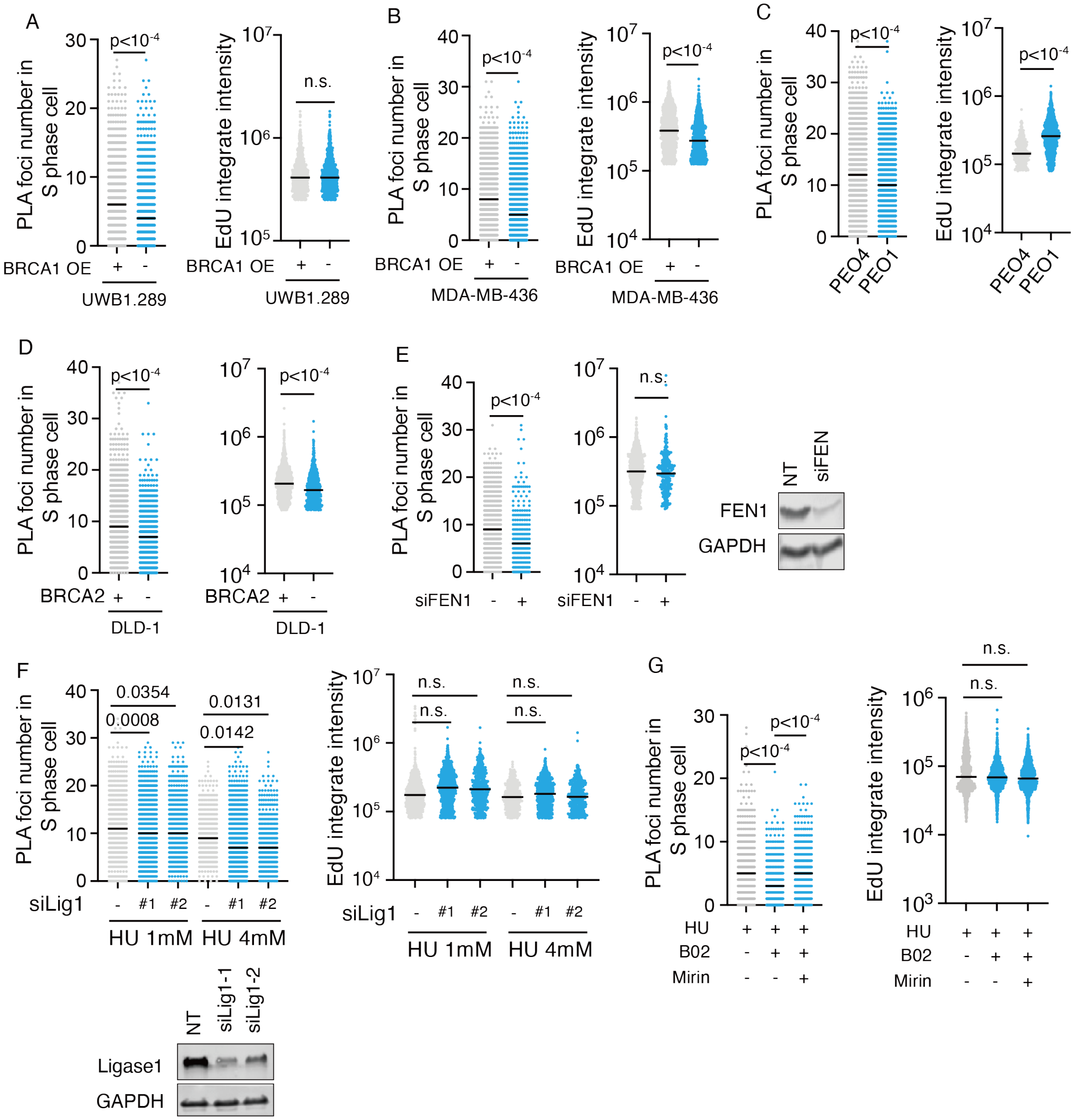


Figure S5. ssDNA gaps loosen the nucleosome assembly on nascent DNA in cells (related to Figure 4).

A–G) PLA for EdU and H3 in indicated treatments. PLA foci number for each cell and EdU intensity as shown (from the same data as Fig. 4). N = 3; p-values from unpaired t-test.

Figure S6 RPA evicts nucleosomes from ssDNA in vitro but does not rescue the low nucleosome density in cells, related to Figure 5

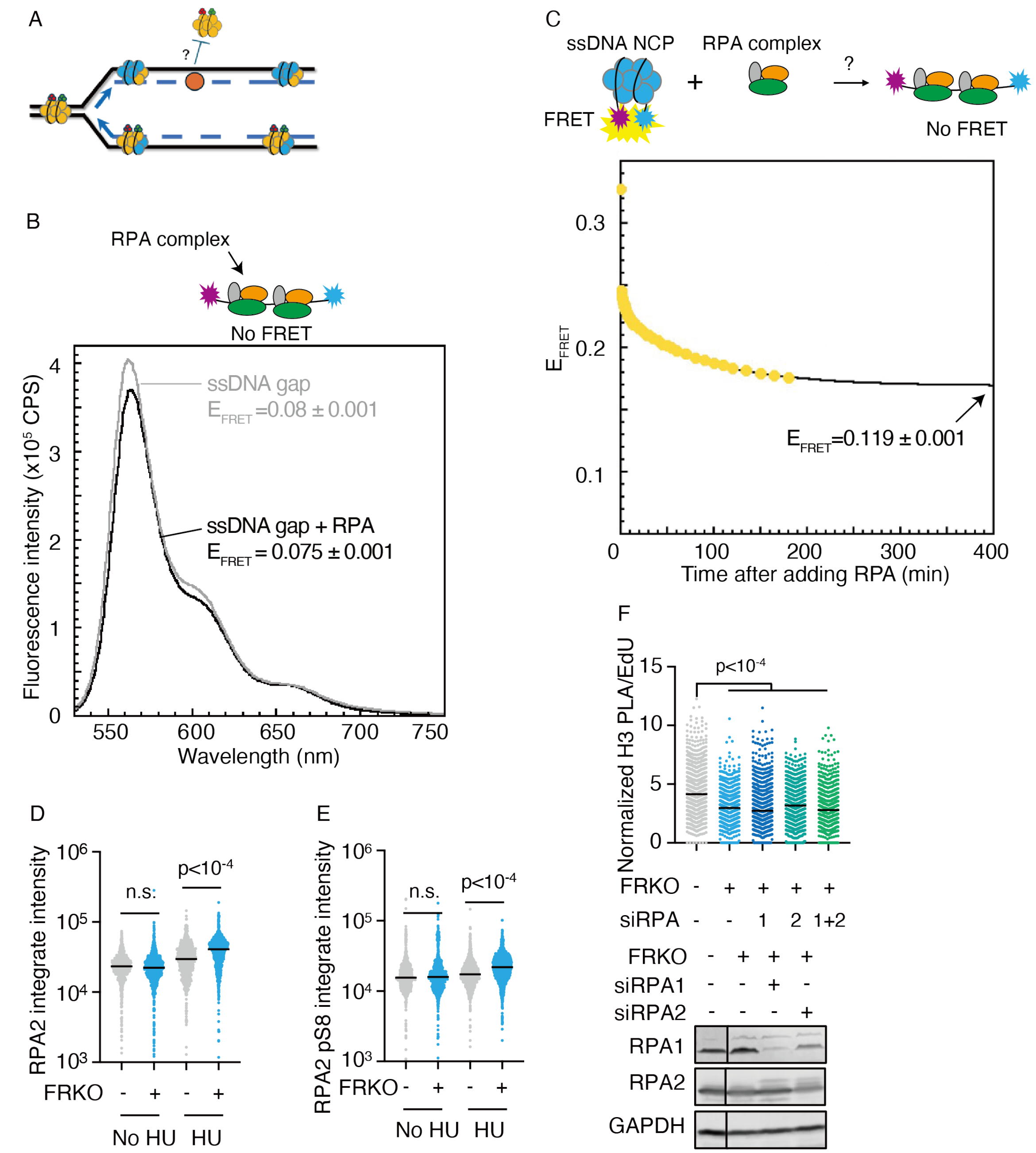


Figure S6. RPA evicts nucleosome from ssDNA in vitro but doesn't not rescue the low nucleosome density in cells

A) Model schematic.

B) FRET signal for ssDNA gap and RPA bound ssDNA gap. Saturating the ssDNA within the ssDNA Gap DNA substrate with RPA does not significantly affect the $E_{FRET}$ observed at equilibrium. Fluorescence emission spectra obtained by excitation at 514 nm is shown. Each spectra is the average of at least two independent spectra with the SEM shown in grey. The average

$E_{FRET}$ values observed at equilibrium for each condition are shown in the inset. Each reported value is the average of at least 4 independent measurements (±SEM).

C) Schematic representation of the FRET pair and assay to monitor unwrapping of ssDNA from around histone octamers within ssDNA Gap nucleosomes. In ssDNA nucleosomes conditions as described in Figure 5E, RPA is added and $E_{FRET}$ values are monitored over time. Trace is the mean of at least three independent traces.

D-E) Immunofluorescence for RPA2 and phosphorylation on RPA2-S8 in WT and FRKO U2OS cells. The cells are treated same as in Figure 6A. N = 3.

F) PLA for EdU and H3 in siRPA1 and/or siRPA2 cells (treated as in Fig. 1B). $N \geqslant 5$; Kruskal–Wallis test. Western blot showed siRNA knockdown efficiency.

Figure S7 Transcription reduces nucleosome density in both WT and FRKO cells

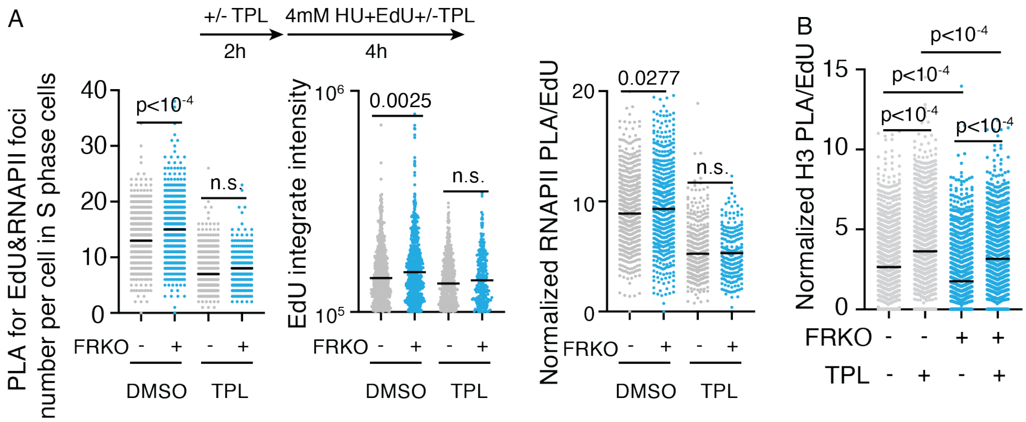


Figure S7. Transcription reduces nucleosome density in both WT and FRKO cells.

A) PLA for EdU and RBP1 in indicated treatments. Left, PLA foci number for each cell and EdU intensity as shown. Middle, EdU integrity intensity for each cell in the indicated treatment. Right, normalized PLA signal in each cell in the indicated treatment. TPL concentration is 10μM. N = 3; p-values from Kruskal-Wallis test.

B) PLA for EdU and H3 in indicated treatments. N = 3; TPL concentration is 10μM. p-values from Kruskal–Wallis test.

Figure S8 HMCES-DPC at ssDNA gaps reduces nucleosome density in FRKO cells, related to Figure 7

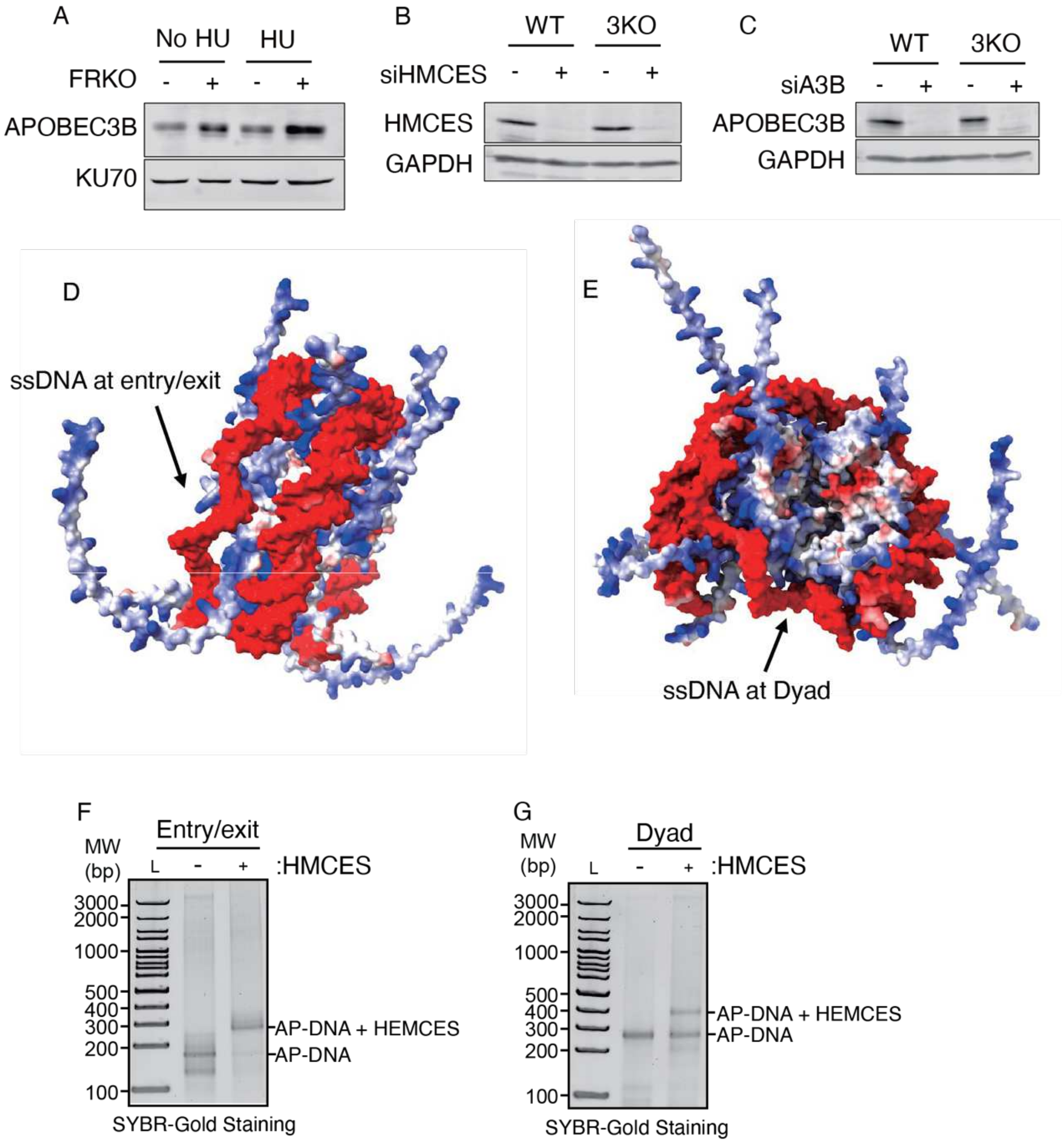


Figure S8. HMCES-DPC at ssDNA gaps reduces nucleosome density in FRKO cells (related to Figure 7).

A) Western blot for chromatin-bound APOBEC3B in WT and FRKO cells with or without HU treatment.

B-C) Western blot for HMCES (B) and APOBEC3B (C) to confirm knockdown efficiency in WT and FRKO cells.

D-E) Structural models of ssDNA-gapped nucleosomes predicted by AlphaFold. D) ssDNA gap at entry/exit. E) ssDNA gap at dyad.

F-G) HMCES-ssDNA crosslink preparation. Gels show HMCES–DNA crosslink products. AP-DNA represents DNA that contains abasic sites; L represents ladder.
